# Phenyl aldehyde and propanoids exert multiple sites of action towards cell membrane and cell wall targeting ergosterol in *Candida albicans*

**DOI:** 10.1186/2191-0855-3-54

**Published:** 2013-09-08

**Authors:** Mohd Sajjad Ahmad Khan, Iqbal Ahmad, Swaranjit Singh Cameotra

**Affiliations:** 1 Environmental Biotechnology and Microbial Biochemistry Laboratory, Institute of Microbial Technology, Sector 39A, Chandigarh 160036, India; 2 Department of Agricultural Microbiology, Aligarh Muslim University, Aligarh 202002, India

**Keywords:** *Candida albicans*, Electron microscopy, Ergosterol, Flow cytometry

## Abstract

In the present study, two phyto-compounds phenyl aldehyde (cinnamaldehyde) and propanoid (eugenol) were selected to explore their modes of action against *Candida albicans*. Electron microscopy, flow cytometry and spectroscopic assays were employed to determine the targets of these compounds. Treatment of *C. albicans* (CA04) with sub-MICs of cinnamaldehyde (50 μg/mL) and eugenol (200 μg/mL) indicated multiple sites of action including damages to cell walls, cell membranes, cytoplasmic contents and other membranous structures as observed under electron microscopy. Concentration and time dependent increase in the release of cytoplasmic contents accompanied with change in extracellular K^+^ concentration was recorded. Exposure of *Candida* cells at 4 × MIC of cinnaamldehyde and eugenol resulted in 40.21% and 50.90% dead cells, respectively as revealed by flow cytometry analysis. Treatment of *Candida* cells by cinnamaldehyde and eugenol at 0.5 × MIC showed 67.41% and 76.23% reduction in ergosterol biosynthesis, respectively. The binding assays reflected the ability of compounds to bind with the ergosterol. Our findings have suggested that the membrane damaging effects of phenyl aldehyde and propanoids class of compounds is attributed to their ability to inhibit ergosterol biosynthesis and simultaneously binding with ergosterol. Indirect or secondary action of these compounds on cell wall is also expected as revealed by electron microscopic studies.

## Introduction

*Candida* species are opportunistic pathogens that cause superficial and systemic diseases in immuno-compromised patients (Richardson 2005). In such persons, infections caused by *Candida albicans* are very common producing oral, vaginal and or systemic candidiasis (Claderone 2002). Candidaisis is the fourth leading blood-borne infection with mortality rates as high as 47% (Pappas et al. 2003) and morbidity rate (30-50%) occurs with systemic *Candida* infections in neutropenic transplant patients (Tortorano et al. 2006). Due to the increasing number of immunosuppressed patients at an unprecedented rate, the management of these fungal infections would be a definite challenge to mankind.

The antifungal drugs most commonly used against these diseases include amphotericin B, ketoconazole, fluconazole, terbinafine and flucytosine. The amphotericin B and azoles targets cell membrane directly or indirectly whereas newly introduced echinocandins target cell wall synthesis (Groll and Kolve 2004; Tortorano et al. 2006). Several current treatments interact unfavorably with other medications, and have resistance problems, a narrow spectrum of activity, limited formulation, toxicity, and also some are fungistatic as opposed to fungicidal (Groll and Kolve 2004). As the population of immuno-compromised patients continues to increase, infectious diseases have become an escalating problem not only in developing countries, but also in the United States and Canada (Sattar et al. 1999). Fungal infections pose a unique problem because both the mammalian host and invading fungi are eukaryotic, making it difficult to develop specific antifungal agents that target selectively only the pathogen.

In these perspectives, there is an increasing demand for novel and effective antifungal agents, justifying the intense search for new drugs from various sources including natural products that are more effective and less toxic than those already in use. Plant products traditionally being used in ethnomedicine as effective antifungal agents, are considered to be a part of the preformed defense system of higher plants (Baby and George 2008; Bakkali et al. 2008) and therefore, expected to deliver active anti-microbial compounds against infectious diseases. The phyto-compounds obtained from many aromatic plants are mixtures of various components (terpenes, aldehydes, alcohols, acyclic esters, etc.) with different chemotypes. They have antifungal effects and their specific anti-*Candida* activity is well known (Knobloch et al. 1989; Khan and Ahmad 2012).

Compounds of chemical groups such as phenyl aldehyde (cinnamaldehyde; 3-phenylprop-2-enal) and phenyl propanoids (eugenol; 4-allyl-2-methoxyphenol) are the major active compounds of cinnamon (*Cinnamomum verum*) and clove (*Syzyzgium aromaticum*) oils, respectively and have shown promising anti-candidal activities (Mehmood et al. 1999; Khan and Ahmad 2012). Yet, the information available regarding the modes of action of these compounds is not sufficient for effective use in chemotherapy. Therefore, an attempt was made to explore the target sites of these two compounds towards *Candida* cells. The electron microscopic studies, flow cytometry, 260 nm absorbing material, extracellular K^+^ leakage and ergosterol biosynthesis and binding assays were performed. Observations of the study have revealed that, in a fungal cell, membranous structures and cell wall are the target sites of these compounds.

## Materials and methods

### Organisms and media

In this study, a clinical strain *Candida albicans* (CA04) isolated from patient of known candidemia attending the Jawaharlal Nehru Medical College and Hospital, Aligarh Muslim University, Aligarh was used. Strain was characterized using morphological and physiological methods such as use of HiCrome Candida agar and Staib’s medium (Himedia Laboratories, Mumbai, India), Dalmau technique, ability to form germ tube and biochemical tests (such as sugar and nitrogen assimilation, urease test and nitrate reduction test), 18 s rRNA gene sequencing and identified as *C. albicans* (Gene bank accession number KC607902). The strain CA04 is deposited in the Microbial type Culture Collection, India (collection number is MTCC 11,802). In addition, a wild type strain of *Escherichia coli* (EC01) was used in ergosterol binding assay. Strain EC01 was isolated from hospital waste water at Jawaharlal Nehru Medical College and Hospital, Aligarh Muslim University, Aligarh and characterized on the basis of its growth on differential media and standard biochemical tests. Strain CA04 was grown in Sabouraud dextrose broth (SDB) and EC01 in nutrient broth (NB).

### Compounds and drugs

The test compounds included in this study were obtained from Himedia Laboratories, Mumbai, India [cinnamaldehyde (minimum assay 98%) and eugenol (98%)]. The drug powders of amphotericin B and fluconazole were purchased from Himedia Laboratories, and Pfizer Co, Mumbai, India, respectively. Stock solutions of amphotericin B and fluconazole were prepared in 1% (v/v) DMSO at a concentration of 25 mg/L and stored at −20°C until used. Compounds were diluted ten-fold in 1% (v/v) DMSO and used in the assays.

### Determination of minimum inhibitory concentration by broth macrodilution assays

The minimum inhibitory concentration (MIC) of drugs was determined against the test strain by CLSI reference method for broth macrodilution M27-A3 (Clinical and Laboratory Standards Institute 2008) and as modified by (Colombo et al. 1995) Briefly, overnight grown culture was adjusted to 0.5 McFarland standards in RPMI 1640 medium with L-glutamine but without bicarbonate and buffered to pH 7.0 with MOPS. 0.1 mL of two-fold serial dilutions of test drugs (10 × concentrations) were made in the test tubes and 0.9 mL of diluted inoculum medium was added to each tubes and incubated at 37°C for 48 h. Drug free control was also included. For the azoles such as fluconazole, itraconazole and ketoconazole, the MIC was established as the lowest concentration that inhibited 80% of the control growth. For amphotericin B, MIC was defined as the lowest concentration that inhibited visible growth. For MIC determination of test compounds, the method of (Sokovic and van Griensven 2006) was adapted with some modifications. Compounds were serially diluted in 1 mL SDB to achieve a range of concentrations from 45 to 800 μg/mL and 10 μL of yeast suspension (0.5 McFarland) was added and incubated at 37°C for 24 h. MIC was defined as the lowest concentration that inhibited visible fungal growth while MFC was the concentration at which no growth was observed. Each experiment was repeated three times and mean values were calculated for MICs and MFCs.

### Time kill assay

Test strain CA04 was assessed for time dependent killing by the test compounds and antifungal drugs namely amphotericin B and fluconazole. Briefly, 2 × MIC concentrations of compounds and drugs were selected for assay. Twenty milliliters of phosphate buffered saline (PBS) solution containing desired concentrations of test agents were inoculated with 1 ml of yeast suspension (0.5 McFarland). The control solution contained PBS with yeast inoculum but no compounds or drugs. Immediately after inoculation 100 μl was collected from the solutions for viable count. Furthermore, test and control solutions were incubated at 37°C for 48 h. Viable counts were obtained from 10-fold serial dilutions of test and control solutions at 2, 4, 6, 8, 12, 24, 36, and 48 h by plating 100 μl of 10-fold dilution onto SDA plates and incubating at 37°C for 24 h. Each experiment was performed in triplicate and the mean colony count for each experiment was converted to values relative to the mean colony count at 0 h to normalize the data and correct the variation in starting inocula concentrations. The relative viable count was plotted against time on a log scale.

### Cellular toxicity assay

The toxicity of compounds was evaluated by the red blood cell (RBC) lysis assay as adapted by (Luiz et al. 2005) with some modifications. The freshly obtained RBCs of sheep blood were washed with 1 mL of phosphate buffer (PB) (pH 7.0) and 4 mL was added to 5% (w/v) glucose solution to obtain 4% RBC suspension. 750 μL of PB containing the desired concentration of test agents was mixed with 750 μL of RBC suspension in Eppendorf tubes and incubated at 37°C for 2 h. Triton X-100 (0.1% (v/v)) in PB was used as a positive control whereas 1% DMSO and PB were used as negative controls. Tubes were centrifuged at 2000 rpm for 10 min and the absorbances of supernatant were read at 540 nm. Percent haemolysis was calculated as: [{(A-B)/(C-B)} × 100]. Where A and B are the absorbance values of supernatant from the test sample and PB (solvent control) respectively and C is the absorbance value of supernatant from the sample after 100% lysis. Each experiment was performed in triplicate and the mean values were considered for calculation of percent haemolysis.

### Determination of effects of compounds on morphology and ultrastructure of fungal cell

#### Scanning electron microscopy

For scanning electron microscopy, 100 μL of cell suspension (0.5 McFarland) was inoculated into 10 mL SDB treated with sub-MICs of cinnamaldehyde (50 μg/mL) or eugenol (200 μg/mL) and incubated at 37°C for 48 h at 120 rpm and harvested to obtain cell pellets. Untreated control was also run. Furthermore, harvested cell pellets were placed into vials containing 3% glutaraldehyde in 0.05 M phosphate buffer (pH 6.8) at 4°C and fixed for 48 h and then dehydrated in an ethanol series (30%, 50%, 70%, and 95%) for 20 min in each alcohol dilution and finally with absolute alcohol for 45 min. The samples were then dried at critical point in liquid Carbon dioxide and mounted on standard ½ inch Cambridge SEM stubs and coated with gold-palladium electroplating (60s, 1.8 mA, 2.4 kV) in a Polaron SEM coating system sputter coater. The samples were examined in a LEO435VP SEM at 15 kV to assess the changes in cell morphology.

#### Transmission electron microscopy

Structural changes produced by test compounds towards fungal cell were evaluated using transmission electron microscopy. Briefly, 10 mL of SDB treated with sub inhibitory concentrations of cinnamaldehyde (50 μg/mL) or eugenol (200 μg/mL) was inoculated with 100 μL of cell suspension (0.5 McFarland) and incubated at 37°C for 48 h at 120 rpm. Control sample did not receive treatment. The obtained cell pellets were fixed with 2.5% glutaraldehyde in 0.1 M cacodylate buffer (pH 7.2) for 24 h at room temperature. Post fixation was carried out in 1% osmium tetroxide in cacodylate buffer. Samples were dehydrated in acetone and embedded in epon. Ultra thin sections were stained with 12.5% alcoholic uranyl acetate and viewed under Morgagni 268D transmission electron microscope at 80 kv. Ultrastructure of treated and untreated cultures was compared to assess the effects of compounds.

### Determination of effects of compounds on fungal cell wall

#### Sorbitol protection assay

Sorbitol assay as described by (Frost et al. 1995) was performed using the broth microdilution procedure. Briefly, duplicate plates (96 well) containing test samples were prepared; one containing two-fold dilutions of test compounds and the other containing test agents plus 0.8 M sorbitol as an osmoprotectant. All the wells were inoculated with 100 μL of cell suspension (0.5 McFarland) plates were incubated at 37°C. The final assay volume was 200 μL. The plates were read at 2 and 7 days. MIC was defined as the lowest concentration that inhibited visible fungal growth and evaluated after 5 and 10 days of incubation. Each experiment was repeated three times in triplicates and mean values were calculated for MICs.

### Determination of effects of compounds on fungal cell membrane permeability and integrity

#### Release of cellular material

Analysis of loss of cellular material absorbing at 260 nm from *Candida* cell was performed using the method of (Bennis et al. 2004) with little modifications. Briefly, *Candida* cells were grown in SDB at 37°C for 48 h and cell suspension (2.5 × 10^7^ CFU/mL) was prepared in 10 mL PB. Furthermore, this suspension was treated with different concentrations (1 ×, 2 ×, and 4 × MIC) of test agents for 1 h or at fixed concentration (4 × MIC) for different time intervals (30, 60, 90 and 120 min). Untreated sample was run as negative control and sample treated with amphotericin B was considered as positive control. After treatment samples were centrifuged at 10,000 rpm for 10 min, and the absorbances of supernatant were read at 260 nm using UV–vis spectrophotometer (UV5704SS, India).

#### Extracellular leakage of potassium

Estimation of extracellular K^+^ leakage from the fungal cell was performed using the method of (Watanabe et al. 2006) with little modifications. Briefly, *Candida* cells were grown in 100 mL SDB at 30°C, 120 rpm for 24 h. Cell pellets were harvested at 5000 rpm for 5 min and washed three times with sterile PB and suspended in 10 mL of PB. 500 μL of the cell suspension was used to determine dry cell weight. 2 mL of cell suspension was mixed with 2 mL of PB containing desired concentration (1 ×, 2 × and 4 × MIC) of test agents and incubated for 4 hr or at a fixed concentration (4 × MIC) for different time intervals (30, 60, 120 and 240 min). Amphotericin B treated cells were used as positive control, whereas untreated cells as negative control. After treatment samples were centrifuged at 10,000 rpm for 10 min, and supernatant was removed and stored for the determination of extracellular K^+^ content released into the medium. The K^+^ concentration was estimated by Flame Photometer (Evans Electroselenium LTD, Halsted Essex, England) using potassium filter.

### Flow cytometry analysis for determination of membrane damage

Flow cytometry analysis was performed to determine the membrane lesion produced by compounds. The method of (Pinto et al. 2006) was adapted with some modifications. Briefly, *Candida* cells (1.0 × 10^6^ CFU/mL) were grown in SDB at 35°C up to mid exponential phase (8 h). The cell suspension was then treated with 1 × MIC, 2 × MIC and 4 × MIC of test agents for 1 h at 35°C. Untreated sample was run as negative control and sample treated with amphotericin B was considered as positive control. The suspensions were centrifuged at 5000 rpm, 5 min and washed three times and re-suspended in PB. The PB suspension was stained with PI (1 μg/mL) at 35°C in dark for 30 min. Unstained cells were always included as auto fluorescence controls. Dead cell associated fluorescence was measured using a FACS Calibur flow cytometer (Beckman Coulter, MOF10, USA) using a blue argon laser at 488 nm. The results were analysed using Sumit 4.3 V software. Flouresence in the FL2 channel (Log red fluorescence, Long pass 630-nm filter) for PI were acquired and recorded, using a logarithmic scale, for 10,000 events per sample. Scattergram analysis was performed to evaluate morphological alterations (size and complexity). For data analysis, quadrants were adjusted in raw data density plots of the fluorescence intensity of control samples in order to include a maximum of 5% of the cells in the upper-right quadrant of plots and then used in the analysis of the remaining samples to quantify the percentage of stained cells in the upper-left quadrant in comparison to agent free controls.

#### Ergosterol quantitation assay

The method of Arthington-Skaggs et al. (1999) with slight modifications was used to determine the effect of test compounds on ergosterol biosynthesis in *Candida* cells. The total intracellular ergosterol amount was quantified in *Candida* cells grown the presence and absence of test compounds. Briefly, 200 μL of *Candida* cells (0.5 McFarland) were inoculated into 20 mL of SDB with and without 1 × MIC, 0.5 × MIC and 0.25 × MIC of test agents and incubated at 35°C for 48 h at 120 rpm. Fluconazole was used as a control. Cells were harvested by centrifugation at 5000 rpm, 5 min and washed once with distill water, dried at room temperature and weight of cell pellet was determined. Further, 3 mL of 25% alc. KOH was added to each pellet and vortexed for 1 min. Suspension was transferred to borosilicate glass tubes and incubated at 90°C for 1 h in water bath and allowed to cool at room temperature. Sterol was extracted by addition of 1 mL of distill water and 3 mL of n-Heptane. Mixture was vortexed vigorously for 3 min and allowed to stand for 30 min. The heptane layer was transferred to clear glass tubes and stored at −20°C for 24 h. Prior to analysis 1 mL of sterol aliquot was diluted 5 fold in 100% ethanol and scanned between 230 to 300 nm using uv-visible spectrophotometer (UV5704SS, India). The presence of ergosterol and the late sterol intermediate 24(2*) DHE in the extracted sample resulted in a characteristic four peaked curve. The absence of detectable ergosterol content in extract was indicated by a flat line. The ergosterol content was calculated as a percentage of the wet weight of the cell by the following equations:

$$\begin{array}{l} \%\;\text{ergosterol}\;+\;\%\;24\left(28\right)\;\mathrm{DHE}=\left[\left\{A_{281.5}/290\right\}\times F\right]\\ \;/\mathrm{pellet}\;\mathrm{weight} \end{array}$$

$$\%\;24\left(28\right)\;\mathrm{DHE}\;=\;\left[\left\{A_{230}/518\right\}\times F\right]/\mathrm{pellet}\;\mathrm{weight}$$

$$\begin{array}{l} \%\;\text{ergosterol}\;=\;\left[\%\;\text{ergosterol}\;+\;\%\;24\left(28\right)\;\mathrm{DHE}\right]\\ \;-\;\%\;24\left(28\right)\;\mathrm{DHE} \end{array}$$

Where, F is the factor for dilution in ethanol and 290 and 518 are the E values (in percentages per centimeter) determined for crystalline ergosterol and 24(28) DHE, respectively.

#### Ergosterol binding assay

### MIC value determination in the presence of ergosterol

To assess the ability of test compounds to bind membrane ergosterol in *Candida* cells, the method as described by (Escalante et al. 2008) was adapted. The MICs of test compounds were determined in the presence and absence of external ergosterol in the growth medium. Briefly, triplicate plates containing test samples were prepared; one containing two-fold dilutions of compounds and the others containing compounds plus ergosterol at a concentration of 100 μg/mL and 200 μg/mL. All the wells were inoculated with 100 μL of yeast suspension (0.5 McFarland) and plates were incubated at 37°C. The final assay volume was 200 μL. The plates were read after 48 h and MIC was determined as the lowest concentration of test agents inhibiting the visible growth.

### Binding assay using centrifugation of intact cells

*Candida* cells were grown to the mid logarithmic phase in 100 mL of SDB. As a negative control wild type *E. coli* strain (EC01) was used and cells were grown to the mid logarithmic phase in 100 mL of nutrient broth. The cells were harvested by centrifugation at room temperature at 3000 rpm for 10 min. The pellets were washed twice with PB and re-suspended in small volume of buffer. A series of 1 mL cell suspensions ranging from an A_600_ of 0 to 4 were prepared. The cells were centrifuged at 3000 rpm for 5 min and re-suspended in the same buffer containing 50 μg/mL of test compounds. As a control, cells were re-suspended in buffer containing no test agents. The cells were incubated for 1 hr, 900 rpm, at room temperature. The amount of unbound compounds in supernatant was determined by measuring absorption at 350 nm.

### Fluorescence measurements of unbound filipin

To evaluate the unbound filipin in competition to binding of membrane ergosterol by eugenol and cinnamaldehyde, the *Candida* cells (0.5 McFarland) were incubated for different time intervals (1, 2, 3 and 4 hrs) in PBS containing 50 μg/mL of cinnamaldehyde/eugenol. Amphotericin B treated cells were used as positive control, whereas untreated cells as negative control. After incubation cells were harvested by centrifugation 3000 rpm for 5 min. The pellets were washed twice with PB and re-suspended in small volume of buffer containing 50 μg/mL Filipin III (Sigma, New Delhi). Filipin is a polyene antibiotic frequently used as a probe for its ability to bind with the membrane ergosterol. After incubation of cells for 1 hr, 900 rpm, in dark at room temperature, the amount of unbound filipin in supernatant was determined by measuring relative fluorescence intensity (excitation at 340 nm and emission at 480 nm) using Synergy H1 Hybrid Reader, BioTek. The reduction in binding of filipin to membrane ergosterol was corresponded to unbound filipin remained in the supernatant when cells were pre-treated with cinnamaldehyde/eugenol compared to untreated cells.

### Statistical analysis

All the experiments were performed three times with three replicates per experiment and data are expressed as mean ± standard deviation. Statistical significance of the differences was determined by the one way ANOVA test using Minitab (V.11.0 for Windows). For cellular mater release experiment, reduction in OD_260_ in the presence of compound was compared to untreated control by one way ANOVA using Duncan’s method. Similarly, extracellular K^+^ leakage in terms of m moles/mg dry weight of cell in treated samples was compared to untreated control. The unbound amount of filipin in supernatant was measured in terms of relative fluorescent intensity of treated samples compared to untreated control. *P*-values of ≤0.05 were considered as statistically significant.

## Results

### MIC of test compounds

MICs of amphotericin B, cinnamaldehyde, fluconazole and eugenol against the test strain were found to be 1.0, 100, 256 and 400 μg/mL respectively.

### Time dependent killing of *Candida* cells

**Figure 1:**
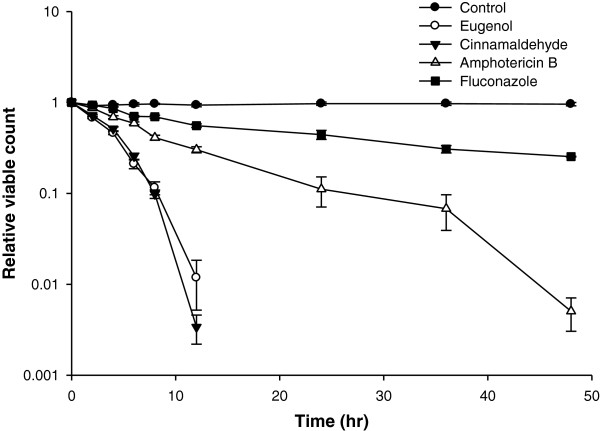
Time kill assay for eugenol, cinnamaldehyde and antifungal drugs against CA04.

The time dependent killing of CA04 by the compounds revealed a decrease of >1log_10_ in the viable count compared to the control at 8 h by cinnamaldehyde and eugenol. Amphotericin B showed a similar effect by 34 h and fluconazole could not up to 48 h (Figure 1).

### Toxicities of compounds to sheep erythrocytes

Test compounds showed no haemolysis at their respective MFCs to test fungi. Only at a concentration two to four times higher of MFCs (2304 μg/mL) was partial haemolysis (10-25%) observed. Similar haemolysis was observed for amphotericin B even at lower concentrations raging from 0.5 to 2.0 μg/mL. Complete haemolysis was shown by 0.1% (v/v) Triton X-100 as a positive control and no haemolysis was exhibited by 1% DMSO and PBS as solvent controls (Table 1).

**Table 1:** Haemolytic property of cinnamaldehyde, eugenol and fluconazole

**Test compounds**	**Percent haemolysis of RBC (Mean ± SD)**							
	**Concentrations of compounds (μg/mL)**							
	**18**	**36**	**72**	**144**	**288**	**576**	**1152**	**2304**
Eugenol	1.65 ± 0.14	1.98 ± 0.09	3.02 ± 0.08	3.72 ± 0.16	4.26 ± 0.16	4.78 ± 0.25	5.60 ± 0.63	19.03 ± 0.30
Cinnamaldehyde	1.84 ± 0.19	2.87 ± 0.07	3.39 ± 0.12	3.96 ± 0.05	4.40 ± 0.17	5.06 ± 0.20	6.22 ± 0.19	18.11 ± 0.40
**Antifungal drug**	**Concentrations of drug (μg/mL)**							
	**0.125**	**0.25**	**0.5**	**1.0**	**2.0**	**4.0**	**8.0**	**16.0**
Amphotericin B	3.56 ± 1.11	8.64 ± 1.89	17.54 ± 2.67	22.45 ± 2.34	31.89 ± 3.22	39.39 ± 3.89	46.11 ± 2.23	58.11 ± 3.10

All the experiments were performed in triplicate and data is presented as mean ± SD.

### Effects of compounds on cellular morphology using scanning electron microscopy

**Figure 2:**
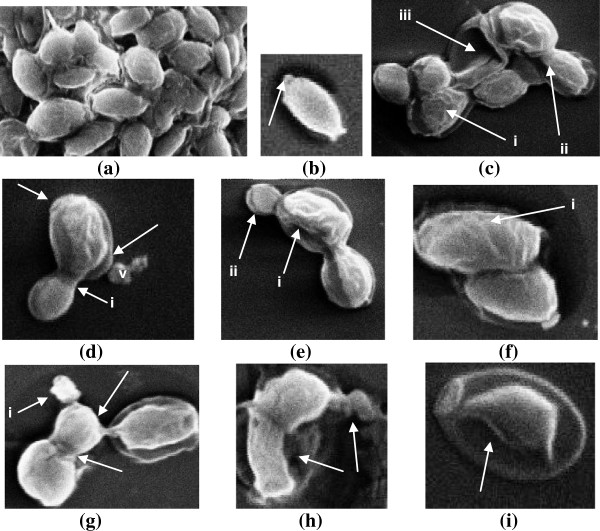
**Scanning electron micrograph of Candida cells treated with cinnamaldehyde (50 μg/mL) and eugenol (200 μg/mL). (a)** untreated; oval shaped and smooth cell surfaces **(b)** untreated; arrow indicates polar bud scar. **(c-e)** treated with cinnamaldehyde; **(c)** shrinkage of cell membrane (i) scar on cell surface indicating lysis of cell membrane (ii) lysed and empty cells (iii) **(d)** deformed interconnected cells (i) with several bud scars without preferential position at the polar ends (arrows) (ii) deposits of lytic material is seen in the form of vesicles (V) **(e)** interconnected cells with convoluted surfaces, loosened cell membrane and receding of cytoplasmic material (i) lysed bud (ii). **(f**-**i)** treated with eugenol; **(f)** deformed cells with convoluted and irregular surfaces (i) **(g)** arrows indicate interconnected cells, lysed cell (i) **(h)** arrow indicates autolysis of cell **(i)** arrow indicates receding of cytoplasmic content from cell membrane and disintegration of cellular content.

The electron micrographs for CA04 obtained from scanning microscopy observations showed important morphological damages (Figure 2). Untreated cells appeared to be oval in shape and with smooth cell surface and polar bud scars (Figure 2a,b). Cells treated with cinnamaldehyde at 50 μg/mL revealed deformed and interconnected cells with shrinkage of cell surface, non polar bud scars and lysis of cytoplasmic material (Figure 2c-e). Similarly treatment with eugenol at 200 μg/mL resulted in deformed cells with convoluted and irregular surfaces. Autolysis of cell and receding of cytoplasmic content from cell membrane was also observed (Figure 2f-i).

### Effects of compounds on ultrastructure of fungal cell using transmission electron microscopy

**Figure 3:**
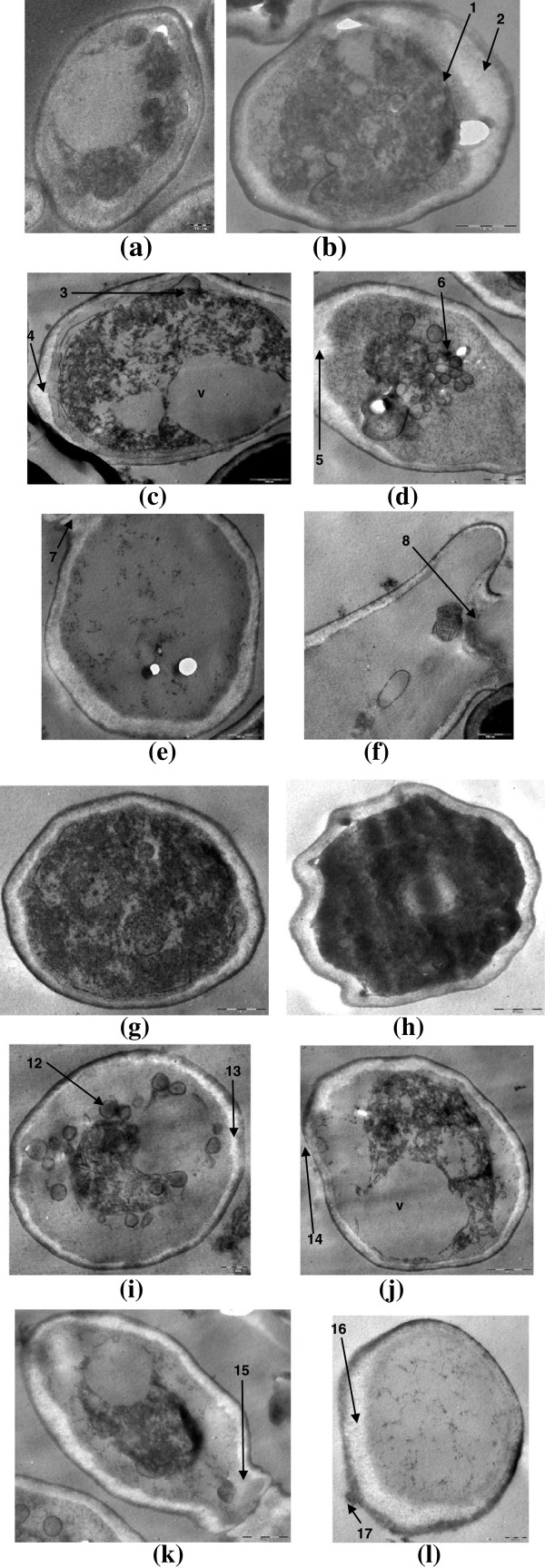
**Transmission electron micrograph of Candida cells treated with cinnamaldehyde (50 μg/mL) and eugenol (200 μg/mL). (a)** untreated; intact cell wall, cell membranes and other organelles. **(b**-**f)** treated with cinnamaldehyde; **(b)** disorganized protoplasm with receding of cell membrane (1), thickening of cell wall (2) **(c)** excessive vacuolization (v), loosening of cell membrane (3), disintegration of cell membrane (4) **(d)** invagination and disintegration of cell membrane (5), deposition of lipid globules **(e)** disintegrated cytoplasm and lysis of cell wall and membrane (7) **(f)** cell wall strength is lost and lysis of cellular material (8). **(g-l)** Treated with eugenol; **(g)** thickening of cell wall (9), loosening of cell membrane (10) **(h)** undulating cell wall (11) **(i)** deposition of lipid globules (12), disintegration of cell membrane (13) **(j)** excessive vacuolization (v), loosening of cell wall and cell membrane (14) **(k)** movement of cytoplasmic material to interconnected cell (15) **(l)** disintegrated cytoplasm, rupturing of cell membrane (16), swelling of cell wall (17).

In untreated sample of *Candida* cells, organelles such as nuclei, mitochondria and nucleus are appeared to be normal (Figure 3a). Treated sample exhibited several changes including thickening of cell wall, stretching of cell membrane, expansion of endoplasmic reticulum, leakage of cell wall and cell membrane, and abnormal distribution of polysaccharides leading to deterioration of cytoplasmic contents (Figure 3b-l).

### Effects of compounds on fungal cell wall integrity

Cell wall damaging effects of an antimicrobial compound can be reversed in the presence of an osmo-protectant compound such as sorbitol. Subsequently MIC of antimicrobial is increased several fold in the test medium containing such compounds. Therefore, to determine the effect of these agents on fungal cell wall, MIC of cinnamaldehyde, eugenol and fluconazole (negative control) and congo red (positive control) was evaluated against CA04 in the presence and absence of sorbitol. As presented in Table 2, MIC of eugenol and cinnamaldehyde was increased up to two-fold against CA04 up to 7 days. Whereas, in the presence of positive control congo red and negative control fluconazole, the MICs were increased up to 512- and two-fold respectively.

**Table 2:** MIC of cinnamaldehyde and eugenol against CA04 in the absence and presence of sorbitol

**Test compounds**	**MIC ( μg/mL)**			
	** 2 days**		** 7 days**	
	**-sorbitol**	**+sorbitol**	**-sorbitol**	**+sorbitol**
Cinnamaldehyde	20	20	40	80
Eugenol	40	40	40	80
**Control**				
Fluconazole	256	256	256	512
Congo red	0.25	16	0.25	128

### Effects of compounds on cell permeability

Effect on cell permeability in terms of cell leakage can be assessed by measuring intracellular component release to the medium from washed cells in buffer. Cellular component which absorb light at 260 nm represent one class of leakage components, primarily nucleotides of which uracil containing compounds exhibit strongest absorbance. The amount of K^+^ leaked out to the external environment is also an indicator of interference with membrane lipid fluidity or integrity. Therefore, to ascertain the effect of these oils onto the cell membrane, the 260 nm absorbing material and K^+^ leakage was determined. Further, these membrane damaging effects were confirmed by flow cytometry.

### Membrane damage in terms of extracellular K^+^ leakage

**Figure 4:**
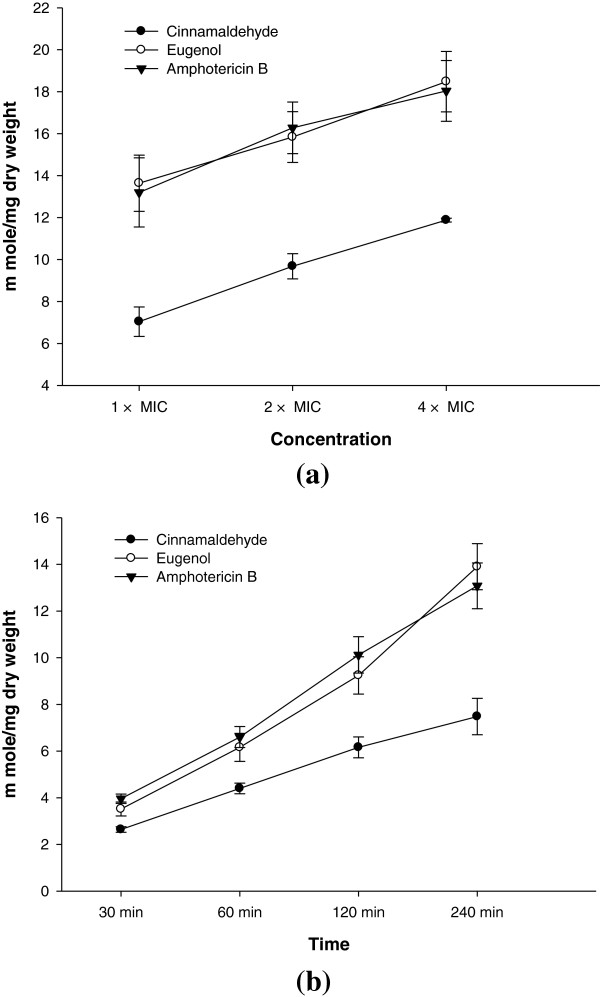
**Effects of cinnamaldehyde, eugenol and amphotericin B on the extracellular K**^**+ **^**leakage in CA04. (a)** concentration dependent **(b)** time dependent.

At 1 × MIC eugenol induced maximum leakage from the *Candida* cells (13.64 m moles/mg dry weight of cell) followed by amphotericin B and cinnamaldehyde. A further increase in K^+^ leakage with increasing concentration for each agent was observed (Figure 4a). Values obtained were statistically significant at *P* ≤0.05. As evident from Figure 4b, the time dependent increase in K^+^ leakage was observed for test agents at 4 × MIC compared to untreated control with the maximum leakage shown by eugenol (13.90 m moles/mg dry weight of cell).

### Release of cellular material

**Figure 5:**
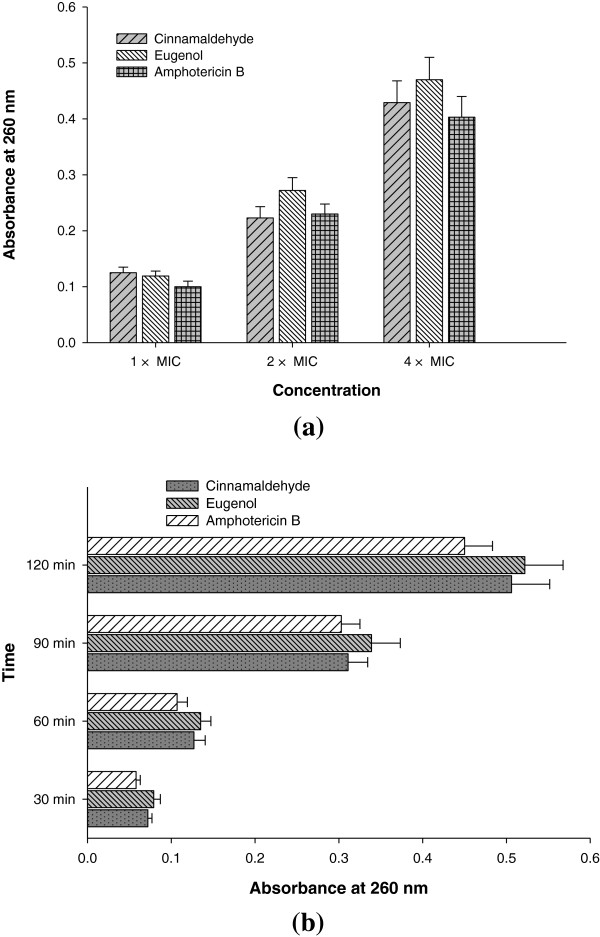
**Effects of cinnamaldehyde, eugenol and amphotericin B on the release of 260 nm absorbing material in CA04. (a)** concentration dependent **(b)** time dependent.

Figure 5a shows increase in the release of cellular content from cells of CA04 in the presence of increasing concentration of test compounds. At 4 × MIC eugenol was most effective (*P* ≤0.05) by exhibiting OD_260_ of 0.470 followed by cinnamaldehyde and amphotericin B (OD_260_ in the range of 0.403-0.429). The treatment with 4 × MIC of test agents induced consistent leakage of intracellular content with the increase in time. Both the compounds and amphotericin B showed significant (*P* ≤0.05) release of cellular material (OD value in the range of 0.450-0.522 at 120 min) compared to untreated control (Figure 5b).

### Membrane integrity assay by flow cytometry

**Figure 6:**
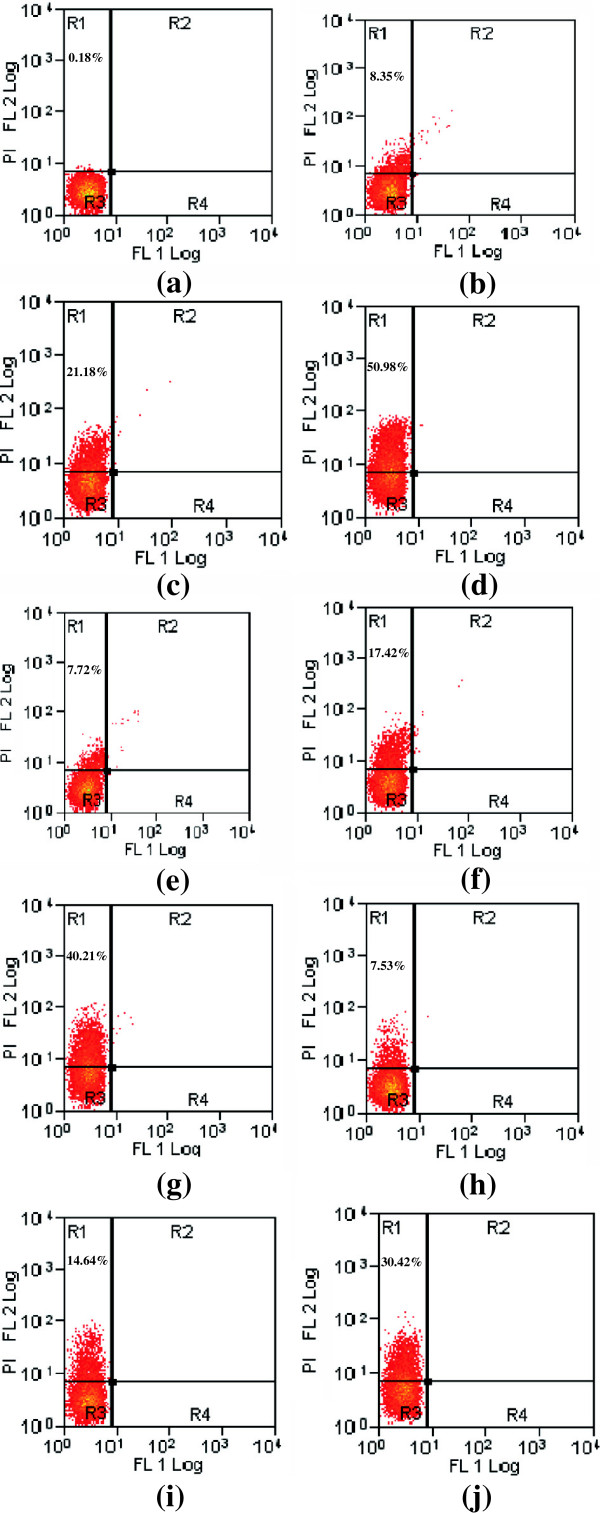
**Membrane lesion produced by test agents in CA04 as analyzed by flow cytometry.** Sequence of density plots are given with respective to percentages of PI-stained cells in upper left quadrant. **(a)** untreated; **(b**-**d)** treated with eugenol at **(b)** 1 × MIC **(c)** 2 × MIC **(d)** 4 × MIC; **(e**-**g)** treated with cinnamaldehyde at **(e)** 1 × MIC **(f)** 2 × MIC **(g)** 4 × MIC; **(h**-**j)** treated with amphotericin B at **(h)** 1 × MIC **(i)** 2 × MIC **(j)** 4 × MIC.

**Figure 7:**
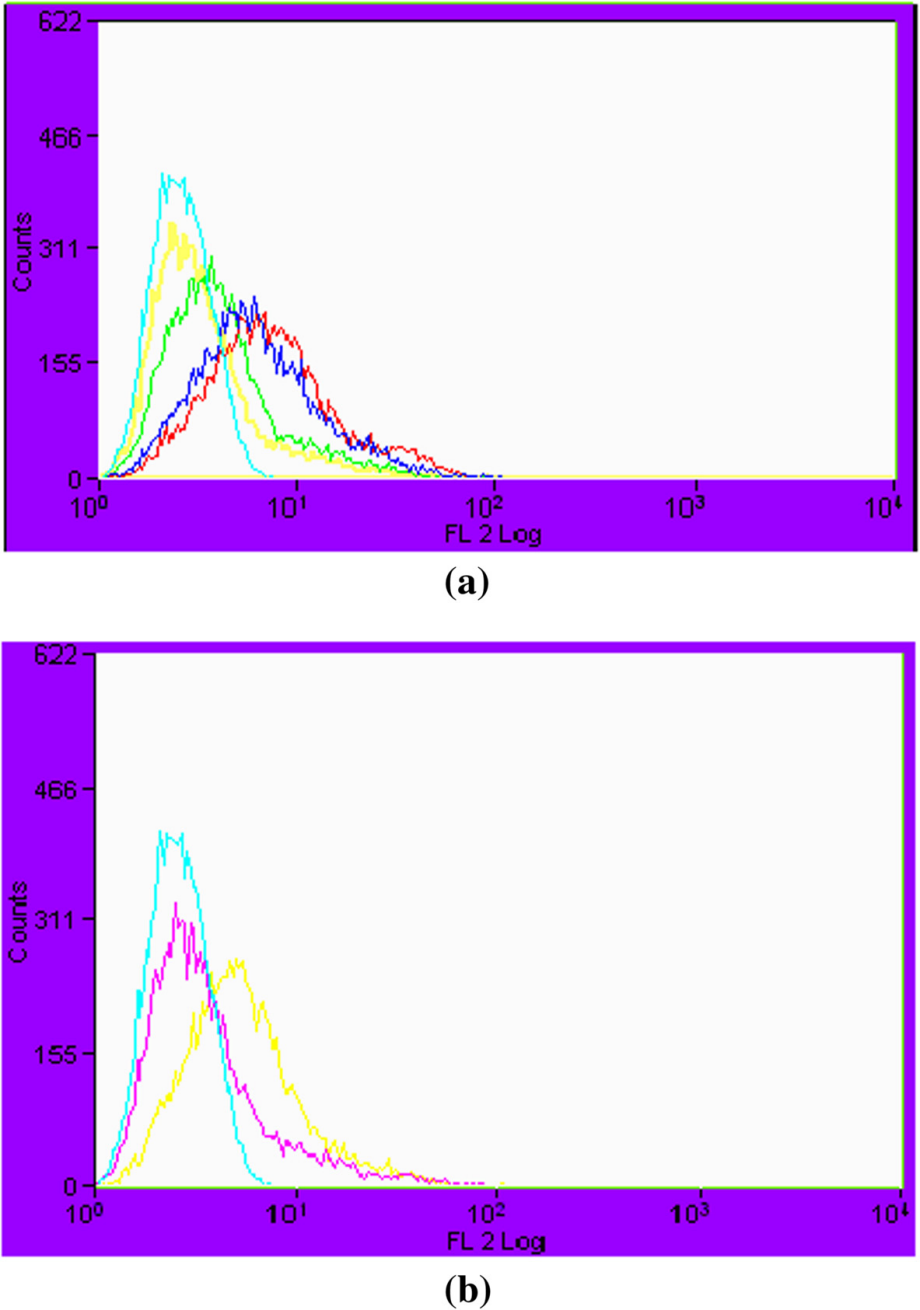
**Histograms showing comparative analysis of viable and non-viable cells in CA04 treated with cinnamaldehyde, eugenol and amphotericin B. (a)** untreated unstained viable cells (red), eugenol at 1 × MIC (dark blue), cinnamaldehyde at 2 × MIC (light green), cinnamaldehyde at 4 × MIC (yellow), eugenol at 4 × MIC (light blue) **(b)** untreated unstained viable cells (red), amphotericin B at 2 × MIC (yellow) and 4 × MIC (light blue).

Propidium iodide is a nucleic acid biding fluorescent probe commonly employed to evaluate the effect of drugs on cell membranes. Cells with sever membrane lesion leading to inherent loss of viability will internalize PI, resulting in an increase in red fluorescence. The PI penetration in *Candida* cells treated with various concentrations of test compounds and positive control is shown in Figure 6. Our results showed eugenol being most effective in damaging the cell membrane of *Candida* cells in treatment of 1 h. At 4 × MIC, 50.98% cells were nonviable Figure 5b-d. Cinnamaldehyde and amphotericn B at this concentration resulted in uptake of PI by 40.21 and 30.42% cells, respectively (Figure 6e-g and h-j). Substantial morphological changes were observed on scattergram of cells upon treatment with test agents. The production of membrane lesion by test agents was increasing with increase in concentration as presented in Figure 7a,b and the efficacy was at par with the membrane damaging effects shown by amphotericin B.

### Ergosterol quantitation assay

Table 3 has summarized the effect of test compounds on ergosterol biosynthesis in CA04 compared to positive control fluconazole. A decrease in ergosterol biosynthesis with the increasing concentration of test agents was observed. Eugenol and cinnamaldehyde were most effective in reducing ergosterol biosynthesis exhibiting 59.61% and 58.96% reduction, respectively compared to untreated control at 0.25 × MIC. At 1 × MIC maximum reduction of 97.07% was recorded for eugenol followed by cinnamaldehyde > fluconazole.

**Table 3:** Ergosterol content of CA04 cells treated with various concentrations of cinnamaldehyde and eugenol

**Test compounds**	**Mean ergosterol content**		
	**0.25 × MIC**	**0.5 × MIC**	**1 × MIC**
Cinnamaldehyde	0.0126 ± 0.001 (58.96)	0.0101 ± 0.001 (67.41)	0.0022 ± 0.0002 (92.84)
Eugenol	0.0124 ± 0.001 (59.61)	0.0073 ± 0.0004 (76.23)	0.0009 ± 0.00006 (97.07)
Fluconazole (control)	0.0257 ± 0.002 (16.29)	0.0197 ± 0.001 (35.81)	0.0118 ± 0.001 (61.57)

Data are presented as a percentage of the wet weight of the cell ± SD, followed in parentheses by the reduction (%) in the mean cellular ergosterol content compared with that of untreated control cell.

Value for untreated control cell was 0.0307 ± 0.003, considered as 100% ergosterol content in an untreated cell.

### Ergosterol binding assay

**Figure 8:**
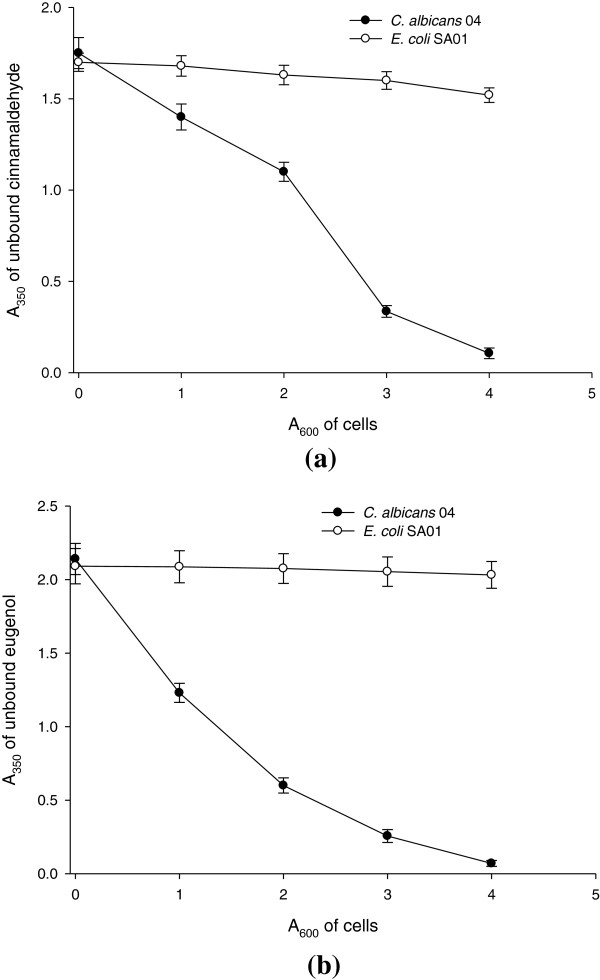
**Binding efficiency of (a) cinnamaldehyde and (b) eugenol to varying cell densities (A**_**600**_**) of *Candida *cells.**

The ergosterol binding assay revealed a two-to four-fold increase in MIC of test agents against CA04 in the presence of 100 μg/mL and 200 μg/mL of ergosterol, respectively (Table 4). This effect was similar to as shown by positive control amphotericin B. Furthermore, the binding of test compounds to yeast cells was studied by measuring the absorbance of unbound compound in supernatant (Figure 8a,b). In addition to test strain, an *E. coli* wild type strains (EC01) was taken as negative control, because it contains no sterols in the plasma membrane. The least amount of binding was observed for this strain, whereas the increasing cell density of CA04 showed increased binding of both the test compounds.

**Table 4:** MIC of cinnamaldehyde and eugenol against CA04 in the absence and presence of ergosterol

**Test compounds**	**MIC (μg/mL)**		
	**-Ergosterol**	**+Ergosterol (100 μg/mL)**	**+Ergosterol (200 μg/mL)**
Cinnamaldehyde	100	200	400
Eugenol	400	1600	1600
Amphotericin B (control)	1.0	2.0	4.0

(−) absence of ergosterol.

(+) presence of ergosterol.

### Fluorescence measurement assay

**Figure 9:**
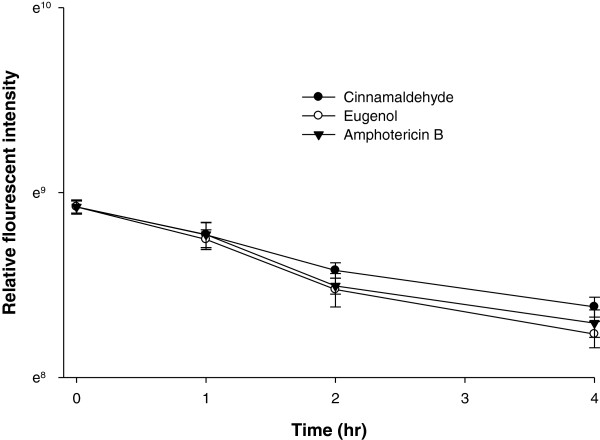
**Relative fluorescent intensity of unbound filipin in suspensions of *Candida *cells treated with cinnamaldehyde, eugenol and amphotericin B.**

Also, the cells pretreated with eugenol or cinnamaldehyde were incubated with ergosterol binding polyene antibiotic filipin to check binding of ergosterol efficacy of eugenol and cinnamaldehyde. The unbound amount of filipin to compound treated cells was determined compared to untreated cells in terms of fluorescent intensity. As shown in Figure 9, the eugenol was found to be more effective in binding (significant at *P* ≤0.05) to membrane ergosterol followed by amphotericin B and cinnamaldehyde.

## Discussion

The commonly used antifungal drugs such as polyenes and azoles are facing problems of host toxicity and either intrinsic or acquired resistance by *Candida* spp (Groll and Kolve 2004). Therefore, there is a need for alternative agents to combat candiadiasis. Plant products have long been used in ethnomedicine and have promised to provide newer and effective antifungal drugs (Knobloch et al. 1989; Bakkali et al. 2008 Mehmood et al. 1999). In spite of well known antifungal properties of phenyl aldehyde and propanoids from plants such as eugenol and cinnamaldehyde, less information is available on their mode of action against *C. albicans*. A better understanding on mode of action of such compounds may provide new insights in combating candidiasis. The present study involved a drug-resistant clinical strain of *C. albicans* (CA04) which was identified on the basis of biochemical and molecular characterization. MICs of amphotericin B, cinnamaldehyde, fluconazole and eugenol against the test strain were found to be 1.0, 100, 256 and 400 μg/mL, respectively. The test compounds were found to be cidal in nature and showed no haemolysis at their respective MFCs to test fungi. Therefore, considering these compounds being fungicidal and non-toxic to mammalian cells the study was planned to investigate their mode of action against the drug-resistant strain of *C. albicans*. In this study we have used electron microscopy, flow cytometry and other assays to explore various sites of action shown by such compounds against *Candida* cells.

To explore the possible mechanism of interaction of compounds with fungal cell wall, membrane and cellular content, strain CA04 was subjected to scanning electron microscopic studies after treatment with sub-inhibitory concentration of cinnamaldehyde (50 μg/mL) and eugenol (200 μg/mL). Untreated cells appeared to be oval in shape and with smooth cell surface and polar bud scars. The cell envelope of *Candida* is appeared to be damaged by the compounds as evident by shrinkage of cell surfaces, presence of non-polar bud scars, and receding of cytoplasm leading to lysis of cells. Increase in buds and bud scars implies that compound affect normal division process of yeasts resulting in a single cell with multiple attempt to divide but not resulting in increase in viable number of cells (Watanabe et al. 2006). The deposition of vesicles on to the cell surface is an indicative of broken cells releasing cytoplasmic materials upon damage of cell wall and therefore, suggests fungicidal nature of compounds towards yeast cells. Similarly, (Bennis et al. 2004) observed the surface alternation induced by thymol and eugenol in *Saccharomyces cerevisiae*. The surface alterations are most probably due to the change in cell permeability showing that the first changes are localized at the plasmamlema and cell wall before any alteration can be detected in the cell interior (De Nollin and Borgers 1974). The indentations of the walls in collapsed cells are indicative of permeability changes that provoke osmotic imbalance.

Further, to ascertain whether sites of action of compounds against *Candida* cell are at cell wall, cell membrane or other intracellular structures, transmission electron microscopy was performed. TEM observations of treated *Candida* cells showed shrinkage of the protoplast, disruption of the cytoplasmic membrane, decomposition of inner organelles, thickening of cell wall, and, even undulant cell wall was observed. Other damages include stretching of cell membrane, expansion of endoplasmic reticulum, leakage of cell wall and cell membrane, and abnormal distribution of polysaccharides leading to deterioration of cytoplasmic contents. An increase in number and expansion of endoplasmic reticula serves to detoxify the drugs or pesticides inside the cell (Park et al. 2007) and indicates a stimulation response of the cell to compounds as evidenced from our findings. Further, accumulation of polysaccharide granules is responsible for the rupture of plasmalemma structure (Ghahfarokhi et al. 2004). These observations indicate that mode of action of such compounds are disrupting the overall intracellular endomembranous system. Therefore, it appears that cell wall and cell membrane integrity, along with other membranous structures are the target sites of these compounds. These effects may be attributed to lipophilic properties of these compounds for their ability to penetrate the plasma membrane (Knobloch et al. 1989). Also, alterations in the cytoplasm and cell organelles may be responsible for the change of cell architecture causing deterioration of the cell envelope. Increase in cell wall thickness of *Candida* cell due to the comparable deposits in the form of electron dense vesicles and or irregular shaped membranous materials were observed in our study. This effect is similar to the fungal cells treated with inhibitors of ergosterol synthesis such as azoles and allylamines which are known to damage membrane because ergosterol is an essential lipid component of the cell membrane (Nishiyama et al. 2005).

Further additional level of evidence on mode of action was assessed by sorbitol protection assay for detection of cell wall as a target site. A distinctive feature of compounds acting on the fungal cell wall is that the antifungal effect can be reversed in a medium containing an osmotic stabilizer such as sorbitol. Subsequently MIC of an antimicrobial is increased several fold in the test medium containing sorbitol. The marginal increase observed in MIC of test compounds was not enough to conclude cell wall as the sole target site by these compounds. Therefore, it may be suggested that primary target of these compounds would not be the cell wall synthesis or assembly. However, the morphological changes observed under electron microscopic studies for the compound treated fungi led to suggest that fungal membrane could be the primary target of these compounds.

Therefore, further experiments to examine effects of compounds on the cell membrane integrity such as determination of extracellular K^+^ leakage reflecting cell membrane damage and assays for UV absorbing material that are released after cell death were also performed. Osmotic stability has been used with *C. albicans* and other fungi to study mode of action of several antibiotics. The amount of K^+^ leaked out to the external environment is also an indicator of interference with membrane lipid fluidity or integrity. Eugenol was most damaging in their effects followed by cinnamaldehyde and amphotericin B. The time and concentration dependent substantial leakage of K^+^ from treated cell has indicated that cytoplasmic membrane is targeted by test compounds and its fluidity and permeability is changed leading to leakage of cytoplasmic material. Dysregulation of ion homeostasis rapidly mediates cell death, forming the mechanistic basis by which a growing number of amphipathic but structurally unrelated compounds such as terpenoid phenols elicit antifungal activity (Zhang et al. 2012). Cellular component which absorb light at 260 nm represent one class of leakage components, primarily nucleotides of which uracil containing compounds exhibit strongest absorbance. Similar to K + leakage, eugenol was most effective in its action. In our study, loss of significant amount of 260 nm absorbing material suggests that nucleic acids were lost through damaged cytoplasmic membrane and is an indicative of gross and irreversible damage to the cytoplasmic membrane by test compounds (Hugo and Longworth 1964).

Effect of eugenol and cinnamaldehyde on to cell permeability was clearly evident from data obtained from K^+^ leakage assays and in 260 nm absorbing materials. This effect was at par with positive control amphotericin B, a membrane disruptive-agent known to affect cell permeability and lethal action against fungal cell. These observations highlight fungal membranes as the sites of action of these compounds. The other workers have also shown the membrane damaging effects of eugenol and cinnamaldehyde but stating mechanisms of action other than those reported in our investigation (Ahmad et al. 2010; Shreaz et al. 2011).

Further confirmation on membrane damages produced by these compounds was done by performing the flow cytometry of treated fungal cells using propidium iodide (PI) as a fluorescent marker. PI is a nucleic acid biding fluorescent probe commonly employed to evaluate the effect of drugs on cell membranes. Cells with sever membrane lesion leading to inherent loss of viability will internalize PI, resulting in an increase in red fluorescence. The results obtained has confirmed eugenol being most effective in a dose dependent manner in damaging the cell membrane of *Candida* cells followed by cinnamaldehyde and amphotericn B. Permeation to PI, particularly following short incubation periods as shown in our study, indicates that the modes of action of these compounds against *Candida* cells involve a primary lesion of the cell membrane with substantial morphological changes leading to cell death.

Since an antifungal agent exhibiting membrane damaging effects could possibly target structural component of membrane i.e. ergosterol, either by lowering the synthesis of ergosterol or by binding with it to disrupt the membrane integrity. Therefore, additional experiments for differentiating these kinds of mechanism, ergosterol quantitation and ergosterol binding assays were performed for *Candida* cells in the presence of test compounds. In our study it was observed that ergosterol biosynthesis was decreased in *Candida* cells when treated with the increasing concentration of test agents. Eugenol and cinnamaldehyde were most effective in reducing ergosterol biosynthesis compared to fluconazole, a well known inhibitor of ergosterol biosynthesis. (Pinto et al. 2009) have also shown ergosterol lowering effects by eugenol but no report is available regarding cinnamaldehyde. Our studies have revealed a dose dependent decrease in the synthesis of ergosterol content at sub-MICs of cinnamaldehyde and eugenol, and the effect was far greater than fluconazole. The effect on membrane integrity, thus, appeared to originate from the inhibition of ergosterol biosynthesis. Further, these compounds were tested for their ability to bind ergosterol. The ergosterol binding assay revealed a sufficient increase in MIC of test agents against CA04 similar to as shown by positive control amphotericin B. These data have led us to assume that compounds are binding with the ergosterol freely available in the medium and hereby amount required to affect the cellular growth has increased.

To further assure that compounds are binding with the membrane ergosterol, the cells were incubated with compounds for a definite period of time and amount of unbound compound in supernatant was detected spectrophotometricaly. In addition to test strain, an *E. coli* wild type strains (EC01) was taken as negative control, because it contains no sterols in the plasma membrane. The least amount of binding of compounds with the membrane ergosterol was observed for this strain, whereas the increase in cell density of CA04 resulted in increased binding of both the test compounds with the ergosterol. The binding of eugenol and cinnamaldehyde with membrane ergosterol was further confirmed in comparison to binding of fluorescent polyene antibiotic filipin. The measurement of unbound filipin in compound treated cells compared to untreated cells has shown that membrane ergosterol is occupied significantly by eugenol and cinnamaldehyde and therefore increase in unbound amount of filipin in the supernatant is observed. In our knowledge there is no report on ergosterol binding ability of these compounds. Here, we have demonstrated that the ergosterol binding ability of eugenol and cinnamaldehyde is an added antimicrobial mode of action from these compounds.

Ergosterol is an important target because it maintains membrane fluidity, asymmetry and integrity and therefore various cell functions (Rodriguez et al. 1985). The compounds, targeting ergosterol that is unique to fungi only, would be selectively toxic to fungi and not to the host cell. Also, the toxicity assay has shown that these compounds are non-toxic to mammalian cells even at higher doses. Ergosterol also contributes to proper functioning of membrane bound enzymes (Lupetti et al. 2002). These compounds primarily exert membrane damaging effects that may be attributed to their ability to lower the ergosterol biosynthesis and simultaneously interacting with membrane by binding to ergosterol. Our findings have highlighted membrane disruption by these compounds and since cell wall synthesizing enzymes are localized in cell membrane, the functioning of these enzymes is also affected. Therefore, partial or secondary action of these compounds is supposed to be cell walls as observed under electron microscopic studies. Not only cell membranes and cell walls but also the other endomembranous structures of cytoplasm are targeted and disrupted by these compounds as evidenced by electron microscopic studies. It would be difficult for a microorganism to overcome these multiple sites of action shown by an antimicrobial agent and hence to develop resistance. Therefore, considering the problem of host toxicity and drug-resistance and realizing the importance and need of alternative agents, our findings suggest that phenyl aldehyde and propanoids class of compounds could be of great value in developing newer drugs.

In the light of the present findings, it could be proposed that antifungal action of such compounds against *C. albicans*, takes place via two step process. The first step involves the passive entry of compounds being ampiphillic in nature into plasma membrane in order to initiate membrane disruption by altering fluidity and integrity because of reduction in the ergosterol synthesis. The second stage is the binding of compounds with the ergosterol and accumulation in the plasma membrane resulting in the inhibition of cell growth due to increased bilayer disorder, leakage of ions and cytoplasmic materials resulting in disorganized cytoplasm. These effects disturb the osmotic balance of cell leading to membrane associated proteins inefficient to perform cellular functions and eventually leading to cell death.

In conclusion, the present results indicate that phenyl aldehyde and propanoids classes of compounds acts by targeting multiple sites in a *Candida* cell including damages to the cell membranes and cell walls. Such effects are attributed to the ability of these compounds to lower the biosynthesis of ergosterol and also to bind it on the membrane. Operation of these two modes of action altogether by such compounds leads to multiple sites of action in *Candida* cells and it could be very useful in combating infections caused by drug-resistant strains. It is suggested that other structurally related compounds from these classes of plant products should be screened to obtain novel and effective antifungal agents against *Candida albicans*.

## Transparency declarations

None to declare.

## Competing interests

The authors declare that they have no competing interests.
